# Extraction of Bioactive Components from *Chamaenerion angustifolium* (L.) Scop. with Choline Chloride and Organic Acids Natural Deep Eutectic Solvents

**DOI:** 10.3390/molecules27134216

**Published:** 2022-06-30

**Authors:** Nikita Tsvetov, Elena Pasichnik, Anna Korovkina, Alevtina Gosteva

**Affiliations:** 1 I.V. Tananaev Institute of Chemistry and Technology of Rare Elements and Mineral Raw Materials—Subdivision of the Federal Research Centre «Kola Science Centre of the Russian Academy of Sciences», Akademgorodok 26a, 184209 Apatity, Russia; angosteva@list.ru; 2 Chemical Department, Apatity Branch of Murmansk State Technical University, Akademgorodok 50a, 184209 Apatity, Russia; wlondr@yandex.ru; 3 Laboratory of Medical and Biological Technologies, Federal Research Centre “Kola Science Centre of the Russian Academy of Sciences”, Fersmana str. 14, 184209 Apatity, Russia; dokktorr@list.ru

**Keywords:** deep eutectic solvents, natural compounds, extraction, chamaenerion angustifolium

## Abstract

*Chamaenerion angustifolium* (L.) Scop. (fireweed) is a perennial herbaceous plant of the Onagraceae family widely used in folk and scientific medicine. It is a promising source of bioactive components. One of the modern trends in extraction is the use of natural deep eutectic solvents (NADESs) combined with ultrasound-assisted extraction (UAE). However, works devoted to the extraction of biologically active substances from *C. angustifolium* using NADESs are scarce. The aim of this work is a comprehensive study of UAE of bioactive components from *C. angustifolium* using NADESs based on choline chloride and malonic, malic, tartaric, and citric acids. The antioxidative properties, total phenols, and flavonoids content were estimated for NADES-based extracts. The reference solvents were water and 90% *v*/*v* ethanol. Volatile extracted components were identified using GC-MS. The kinetics of the UAE were studied at 45 °C for 20–180 min with water added to 30 wt% NADES. The power of the ultrasound was 120 W, and the frequency was 40 kHz. It was found that NADES choline chloride + citric acid is more effective for the extraction of bioactive components. For this, NADES UAE conditions were optimized following a Box–Behnken design of the experiment and a response surface methodology. The temperature ranged from 30 to 60 °C, the time of extraction ranged from 20 to 60, and the addition of water ranged from 30 to 70 wt%. We established the optimal extraction conditions: temperature 58 °C, time of extraction 35 min, and 70 wt% water. The obtained results expand the knowledge about the use of NADES for the extraction of biologically active compounds from cheap and available plant raw materials.

## 1. Introduction

*Chamaenerion angustifolium* (L.) Scop. (fireweed) is a perennial herbaceous plant of the Onagraceae family. This plant is widely distributed throughout the northern hemisphere, including large parts of the boreal forests. Fireweed is a widely used folk medicine plant; it also is used for drinks and honey production. Due to folk medicine, this plant improves genitourinary system action, stimulates immunity, prevents viral diseases, and increases vitality [1]. It contains several bioactive compounds such as phenolic acids, cinnamic acid derivatives, ascorbic acid, flavonoids (quercetin, kaempferol, and myricetin derivatives), tannins, coumarins, carotenes, sterols, and triterpenes; they provide a high pharmacological activity such as antioxidant, antibacterial, anti-inflammatory, analgesic, and anticancer properties [2,3,4,5,6]. The content of flavonoids in this plant is one of the highest among the herbaceous plants of the Kola Peninsula [7]. *C. angustifolium* quickly accumulates a large amount of biomass and is quite easy to cultivate, which makes it a valuable potential source of nutrients and raw materials for further processing. Thus, the works devoted to the extraction of useful substances from this plant are quite relevant. This requires a thorough study of both the biochemical composition of *C. angustifolium* and using new solvents to extract biologically active substances.

One of the modern approaches to the extraction of bioactive components is the use of deep eutectic solvents (DES). They are a mixture of a hydrogen bond donor (HBD) and a hydrogen bond acceptor (HBA). The mixtures are characterized by a very large difference between the melting point of the mixture and the individual components. These mixtures were described for the first time by A. Abbott in the early 2000s [8]. HBA and HBD are usually solid at room temperature, but their mixture is liquid at the eutectic point. Carboxylic acids, sugars, polyols, inorganic salts, etc., can act as HBDs; quaternary ammonium bases, i.e., choline chloride and tetrabutylammonium chloride, can act as HBAs. Later, in 2011, it was proposed to separate mixtures containing dicarboxylic acids, amino acids, and sugars into a separate subclass, natural deep eutectic solvents (NADES) [9]. DESs are similar to ionic liquids, but, unlike the latter, they are non-toxic, biodegradable, and low-cost. Unlike conventional organic solvents, DESs are non-volatile and inflammable. Due to the presence of these remarkable properties, DESs are being actively studied; they have already found applications in various fields, such as organic synthesis, electrodeposition, analytical chemistry, and the extraction of bioactive components [10,11,12,13,14]. DESs successfully extract polyphenolic compounds and other bioactive components from plant material [10,15,16].

Ultrasound-assisted extraction (UAE) is actively used for the extraction of bioactive components from plants using DESs [11]. In contrast to conventional extraction techniques (maceration, Soxhlet extraction, heat reflux extraction, etc.), UAE is a time-effective and environmentally friendly extraction method. The extraction is accelerated due to plant tissue rupture caused by the cavitation, formation, and collapse of bubbles. Moreover, an acceleration of extraction is more important for viscous solvents, i.e., NADES. Recently, this technique was successfully used for the extraction of polyphenols and flavonoids from various plant samples [17,18,19]. The mixtures of choline chloride and organic acids, such as malic and citric acid, are considered promising solvents for extracting the substances mentioned above [20,21,22,23]. The study of UAE includes kinetic modeling and the optimization of extraction conditions, in particular the assessment of the effect of influence of the temperature, the nature of the solvent, the solid-to-liquid ratio, etc. Unfortunately, not many works are devoted to the detailed research and modeling of the kinetics of UAE.

However, at the moment, works devoted to the extraction of biologically active substances from *C. angustifolium* using NADES are scarce. There are no data on which composition of NADES and which extraction conditions are optimal for obtaining bioactive components from this plant. Moreover, there are few works devoted to the study of the phytochemical composition of extracts and the content of volatile components in them. Thus, the aim of this work is a comprehensive study of the extraction of bioactive components (polyphenols, flavonoids, and antioxidants) from *C. angustifolium* using NADESs formed by choline chloride and malonic, malic, tartaric, or citric acids, the optimization of extraction conditions, and the identification of the volatile components extracted with various solvents.

## 2. Materials and Methods

### 2.1. Collection and Preservation of Plant Material

The leaves of *Chamaenerion angustifolium* (L.) Scop. were collected in the flowering vegetation period (in mid-August 2020) in the woodland edge near Apatity (Murmansk region, Russia) during a blossoming phase. The plant material was dried in air at 25 °C for a week (until mass stabilization) and stored in accordance with [24]. Air-dried plant material was powdered with a blade grinder and sieved with laboratory sieves with 0.1–0.5 mm openings.

### 2.2. Chemicals and Reagents

The study included the application of the following reagents: choline chloride (99%, Rongsheng Biotech, Xi’an, China), malonic, malic, tartaric, and citric acids (>99%, Vekton, St. Petersburg, Russia), 2,2-diphenyl-1-picrylhydrazyl (99%, Sigma-Aldrich, Burlington, MA, USA), Folin–Ciocalteu reagent (2M;, Sigma-Aldrich, Burlington, MA, USA), ammonium molybdate, potassium dihydrogenphosphate, aluminum chloride (>99%, Vekton, St. Petersburg, Russia), concentrated sulfuric acid (>94%, Nevareactiv, St. Petersburg, Russia), gallic acid (98% Sigma-Aldrich, Burlington, MA, USA), rutin (≥94%, Sigma-Aldrich Burlington, MA, USA), ascorbic acid (>99.7%, Hugestone, Nanjing, China), ethanol (96%, RFK Company, Moscow, Russia), and deionized water obtained with a “Millipore Element” water purification system (Millipore, Darmstadt, Germany).

### 2.3. Preparation of NADESs

Choline chloride served as an HBA. It was mixed with the appropriate amount of malonic (MA), malic (Mal), or citric (CA) acids as the HBD at a molar ratio of 1:1 or with tartaric (Tar) acid at a molar ratio of 2:1, as stated in [13]. The reagents were weighed by an Acculab Atilon laboratory balance (Sartorius group, Goettingen, Germany). The mixtures were heated at 80 °C for several hours to obtain homogeneous liquids. NADESs are highly viscous liquids, they may be dissolved with water to decrease the viscosity [25]. In our work, 30–70 wt% water was added to NADESs. The prepared NADESs were characterized using the ^1^H NMR method with a 500 MHz Bruker AVANCE III NMR spectrometer (Bruker, Billerica, MA, USA) equipped with a BBI probe head with an inner coil for 1H nuclei and using the FTIR method with a Bruker Alpha FT-IR spectrometer (Bruker, Billerica, MA, USA) with a Platinum ATR attachment, as described in our previous work [26].

### 2.4. Ultrasound-Assisted Extraction and Optimization Using Box–Behnken Design

**Figure 1:**
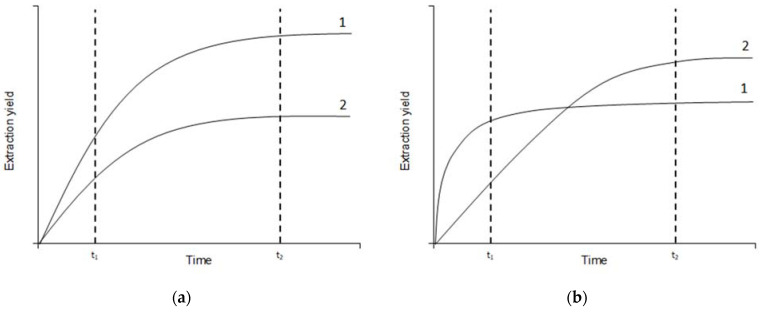
Theoretically possible mutual arrangements of kinetic curves of two solvents. (**a**) Curves do not cross, (**b**) curves do cross.

In order to choose the most suitable NADES for further work, it was necessary to compare the efficiency of extraction of biologically active components with various solvents. However, in order to properly compare the efficiency of the solvents, it was necessary to get close enough to achieving phase equilibrium, which meant we had to evaluate the kinetics of extraction. Theoretically, in dependence on the solvent nature, two cases are possible: the kinetic curves do not cross (Figure 1a) or they do cross (Figure 1b). In case (a), the extraction yield for solvent 1 is higher than that for solvent 2 all the time (both at times *t*_1_ and *t*_2_), and the curves do not cross. In case (b), the curves do cross. At the beginning of the extraction (*t*_1_) the extraction yield for the solvent 1 is higher than that of solvent 2, but as the phase equilibrium is achieved (*t*_2_), the extraction yield for solvent 2 is higher than that of solvent 1. It is important to know when the phase equilibrium is achieved so that we can adequately compare the extraction efficiency of the different solvents. Thus, it is important to evaluate the extraction yield at the correct extraction time.

Extraction was described in detail in [26]. Briefly, the ratio of plant material and the solvent was 1:10 (*w*/*v*). UAE was performed in the VBS-3DP thermostated ultrasound bath (Vilitech, Moscow, Russia) with an ultrasound power of 120 W and an ultrasound frequency of 40 kHz. After extraction in 1.5 mL Eppendorf tubes, the samples were centrifuged at 4000 rpm for 5 min. The extraction kinetics were researched at 45 °C using NADESs with 30% water. The reference solvents were water and 90% ethanol. The extraction times were 20, 40, 60, 120, and 180 min. We determined that the best time is 60 min.

The optimization of extraction was carried out by the Box–Behnken design of experiment with three levels of three parameters. Due to the method, the central point was replicated five times. The parameters and their levels are presented in Table 1. The temperature limits were chosen to be 30–60 °C following our previous work [26]. The addition of 30% by weight of water to the studied NADES was chosen as the minimum additive since a smaller additive leads to a significant increase in viscosity, which makes it difficult to work with the extragent. The maximum water addition was 70 wt%.

### 2.5. Chemical Analysis

All chemical analyses are described in detail in [26]. The total phenolic content (TPC) was determined by a reaction with a Folin–Chocalteu reagent [27]. The total flavonoid content (TFC) was determined using the complexation reaction with aluminum chloride [7]. For these analyses, row extracts were diluted 100 times. The total antioxidant capacity (TAC) was estimated using the phosphomolybdate method [28]. For the TAC, 5 μL of raw extract was mixed with 2 mL of reagent solution without additional dissolving. Free radical scavenging (FRS) was estimated with the DPPH method [23] for extracts diluted 400 times.

The calibration curves for TPC were prepared using solutions of gallic acid (10–200 μg·mL^−1^) and were expressed as mg/g of gallic acid equivalent (GAE) per one gram of plant weight; curves for TFC used rutin (100–1000 μg·mL^−1^) and were expressed as mg/g of rutin equivalent (RE) per one gram of plant weight; and curves for TAC used ascorbic acid (1.25–10 mg mL^−1^) and were expressed as mg/g of ascorbic acid equivalent (AAE) per one gram of plant weight. It should be particularly noted that calibration curves were obtained for each type of solvent, and it was established that the nature of the solvents influences the parameters of the linear regression.

### 2.6. GC/MS Analysis

The components of extracts were analyzed with a GCMS-QP2010 (SHIMADZU, Kyoto, Japan) equipped with an HP-5MS column (Agilent J&W, Santa Clara, CA, USA) with a (5%-phenyl)-methylpolysiloxane phase (film thickness 0.25 μm), length 30 m, internal diameter 250 μm. Helium was used as the carrier gas at a flow rate of 1 mL/min. The injector temperature was set at 280 °C. The temperature program was as follows: isothermal step at 40 °C for 3 min, ramp 40 to 280 °C with a heating rate of 10 °C/min, and isothermal step at 280 °C for 5 min. Thus, the total running time was 32 min.

For the gas-chromatography analysis, 0.1 mL of water-based or NADES-based extracts were mixed with 1 mL of hexane and were intensively shaken for a few minutes. After this, the hexane phase was analyzed. Ethanolic extracts were injected into the chromatographic system directly. The aliquot volume was 1 μL. It was injected in split mode 1/10 for water-based and NADES-based extracts and 1/80 for ethanolic extracts.

Each of the peaks of the mass spectra were compared with the NIST 27.147 database. Since no internal standard was included, only compounds with similarity indices of 80% and above were taken into account.

### 2.7. Kinetical Analysis

The extraction kinetics data were approximated by a second-order model according to [29,30]. Such an approach was successfully applied to describe extraction kinetics in NADES [31]. According to a second-order model, the dependence of the concentration ($Y_{t}$) is expressed in terms of the equilibrium concentration (Y(eq)) and the rate constant (*k*):

(1) $$Y_{t}=\frac{kt{\left(Y\left(eq\right)\right)}^{2}}{1+ktY\left(eq\right)}$$

The parameters *k* and Y(eq) can be found form the linearized Equation (1) in the coordinates tYt vs. *t*:

(2) $$\frac{t}{Y_{t}}=\frac{1}{Y\left(eq\right)}t+\frac{1}{k{\left(Y\left(eq\right)\right)}^{2}}$$

Thus, the equilibrium concentration is found from the slope of the line, and the rate constant is found from the shift of the line.

### 2.8. Statistical Analysis

The measurements for the comparison of extraction effectiveness were made three times for each analysis. The statistical comparison was performed using a factorial analysis of variance (ANOVA) and a post-hoc Tukey’s HSD test. These two methods were used to estimate statistically significant differences at *p* ≤ 0.05. The calculations were performed using MS Excel 2010 (Microsoft, Redmond, DC, USA) with the Real Statistics Resource Pack add-on [32]. For the Box–Behnken design of the UAE condition optimization, an ANOVA test and a response surface methodology were used via DesignExpert 11 (Stat-Ease, Minneapolis, MN, USA) software.

## 3. Results and Discussion

The results of the kinetic parameters estimation are presented in the Table 2. The higher the value of a rate constant, the lower the equilibrium achieving time. Among the TPC, the highest rate constant was observed for ethanol (2.5×10^−3^ g·mg^−1^·min^−1^), and the lowest was observed for NADESs (0.6–1.2 × 10^−3^ g·mg^−1^ min^−1^). This may be partly related with the fact the NADESs are more viscous than ethanol. However, it may be noted that the rate constant for water was lower than for NADES choline chloride + malonic acid. For the TFC, the highest rate constants were obtained for ethanol (2.7 × 10^−3^ g·mg^−1^·min^−1^) and NADES choline chloride + citric acid (3.6 × 10^−3^ g·mg^−1^·min^−1^). For the TAC, the rate constant for water (144.6 × 10^−3^ g·mg^−1^·min^−1^) was much higher than for other solvents. The rate constants for DPPH were very different for different solvents. The highest values were obtained for water (155.6 × 10^−3^ g·mg^−1^·min^−1^) and NADES choline chloride + tartaric acid (186.4 × 10^−3^ g·mg^−1^·min^−1^), and the lowest one value was obtained for NADES choline chloride + malic acid (2.3 × 10^−3^ g·mg^−1^·min^−1^). Generally, it may be noted that the rate constants for TAC and DPPH were much higher than for TPC and TFC, which may indicate that the antioxidant and antiradical activities do not only depend on the polyphenol or flavonoid contents in extracts. Some substances responsible for antioxidant properties were extracted faster than the polyphenolic compounds.

**Figure 2:**
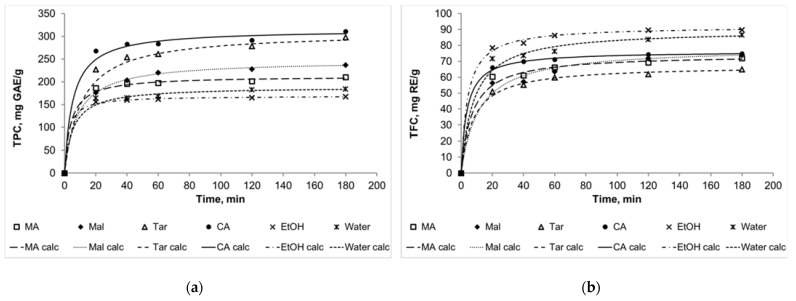

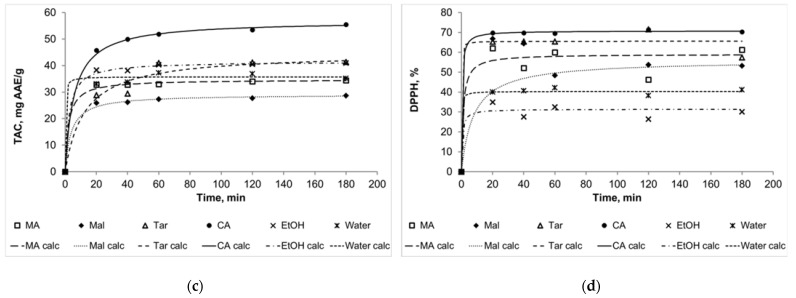
The kinetic curves for TPC (**a**), TFC (**b**), TAC (**c**), and DPPH (**d**). MA: NADES choline chloride + malonic acid, Mal: NADES choline chloride + malic acid, Tar: NADES choline chloride + tartaric acid, CA: NADES choline chloride + citric acid, EtOH: 90% (*v*/*v*) ethanol; “calc” means calculated data.

The kinetic curves for TPC, TFC, TAC, and DPPH (Figure 2) show a good quality of approximation. Moreover, it should be noted that in some cases the curves for several solvents intersect, and this was mentioned above as the theoretically assumed case (Figure 1b). For example, the curve for NADES choline chloride + malic acid intersects the curve for NADES choline chloride + malonic acid in the case of flavonoid extraction kinetics. In general, we can assume that 60 min is enough time for the main part of the extraction to be completed and the system to approach the state of equilibrium. Extracts obtained during this time can be compared according to the analyzed parameters to assess the effectiveness of certain solvents.

**Figure 3:**
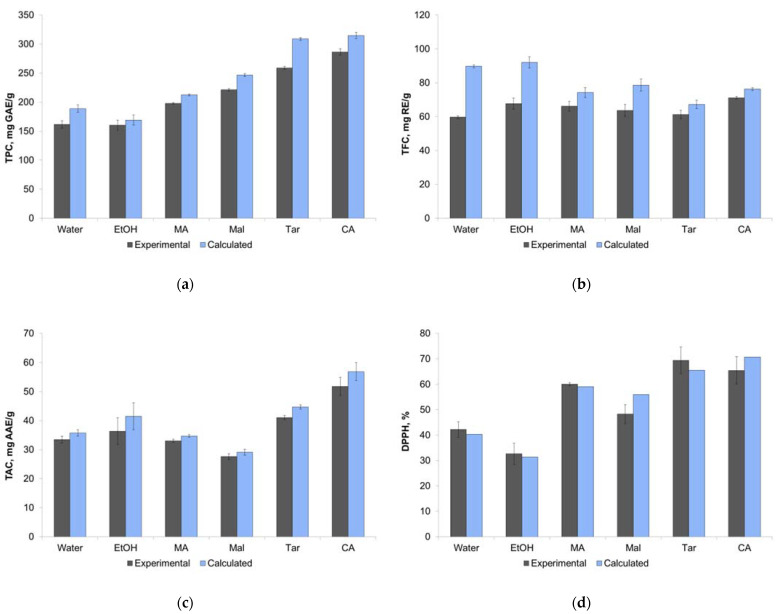
Comparison of extraction efficiency from experimental and calculated data for different solvents by parameters TPC (**a**), TFC (**b**), TAC (**c**), and DPPH (**d**). Data are presented with ± SD of three repetitions.

Thus, the effectiveness of different solvents was compared after 60 min of extraction. Moreover, using the calculated equilibrium values of TPC, TFC, TAC, and DPPH, it was possible to compare the experimental and calculated effectiveness of the different solvents. Figure 3 presents the experimental values of TPC, TFC, TAC, and DPPH for 60 min of extraction (± SD) are along with the calculated values. Despite the calculated equilibrium values being a little higher than the experimental ones (because 60 min is, strictly speaking, not enough time for the full equilibrium setting), the general trend is the same for both types of data. The results for TFC can be considered as the exception: the calculated equilibrium values for ethanol and water were higher than for NADESs.

The values of TPC in the case of NADESs choline chloride + citric and tartaric acids reached 250–300 mg GAE mL^−1^, which was almost twice as much as for ethanol and water (near 150 mg GAE mL^−1^). The TFC values for all solvents were near 60–70 mg RE mL^−1^, and it may be concluded that flavonoids are extracted from this plant equally well with water or ethanol and NADESs. The highest TAC value was obtained for NADES choline chloride + citric acid (above 50 mg AAE mL^−1^). The DPPH values for NADESs were higher than for ethanol and water, and again, the highest values were obtained for NADESs choline chloride + citric and tartaric acids.

The ANOVA and the Tukey’s HSD test (*p* < 0.05) were carried out to evaluate the differences between solvents (Table 3). It was observed that the TPC values were significantly different for all pairs of solvents, with the exception of the ethanol–water pair. The differences for TFC values were insignificant for most solvent pairs, with the exception of the pairs of NADESs choline chloride + citric acid and malic acid, water, and ethanol–water. For the TAC, there were no differences between ethanol, water, and NADESs with malonic or malic acids, but NADESs with citric and tartaric acids showed higher TAC values. For the DPPH values, ethanol and water showed equal effectiveness, as did NADESs with citric, malonic, and tartaric acids.

Thus, the most promising NADES for further investigations is choline chloride + citric acid. Since it was found from kinetic experiments that the main part of the extraction takes place within an hour, this time was chosen as the limit in the optimization experiments.

Following the response surface methodology, the responses were described with polynomial equations. The second-order response surface model was chosen as the most suitable for all responses types; the parameters were found by experimental data approximation:

TPC = 266.68 + 7.85A − 0.37B + 16.262C + 1.15AB + 0.13AC − 2.38BC + 1.45A^2^ + 6.1B^2^ + 9.68C^2^ (3)

TFC = 62.01 + 2.13A − 1.71B + 5.89C − 0.33AB + 0.13AC − 1.18BC − 1.61A^2^ + 4.45B^2^ + 4.25C^2^ (4)

TAC = 43.98 + 1.73A − 2.83B + 4.25C − 0.80AB − 0.42AC − 1.20BC + 0.91A^2^ + 3.63B^2^ + 2.46C^2^ (5)

DPPH = 65.73 + 2.03A + 4.02B + 1.17C − 0.75AB + 1.08AC + 1.65BC + 2.21A^2^ − 2.87B^2^ + 0.56C^2^ (6)

where A is the temperature, B is the time, and C is the water content in NADES.

The results of the ANOVA test (Table 4) demonstrate a good approximation for TPC and TFC but not for TAC and DPPH. This may be due to the fact that the TAC parameters depend on different groups of substances and the overlap of the different extraction processes leads to more complex dependencies. For TPC, A, C, and C^2^ are significant model terms; for TFC, C, B^2^, and C^2^ are significant model terms; for TAC, C and B^2^ are significant model terms; and for DPPH there are no significant model terms. The value of R^2^ was 0.8961 for TPC, 0.8270 for TFC, 0.7679 for TAC, and 0.6166 for DPPH. The relatively high *p*-values for the “Models” term and their insignificance for TAC and DPPH may be a common tendency for these extract properties. Similar results can be found in [33].

**Figure 4:**
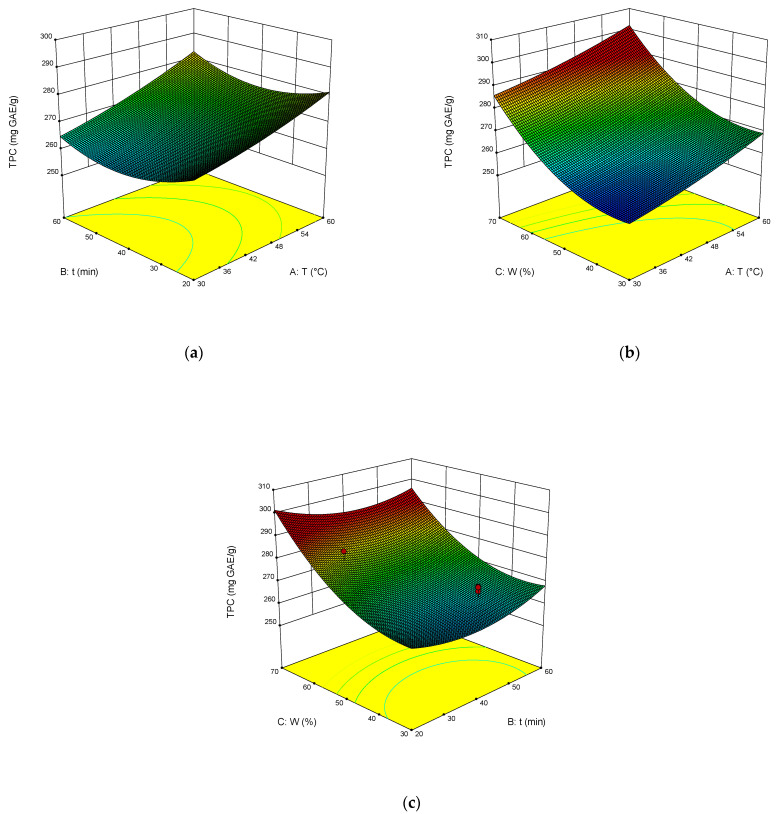
Response contour plots (**a**–**c**) showing the extraction temperature (A), extraction time (B), and water content (C) effect on the extraction yield of TPC.

**Figure 5:**
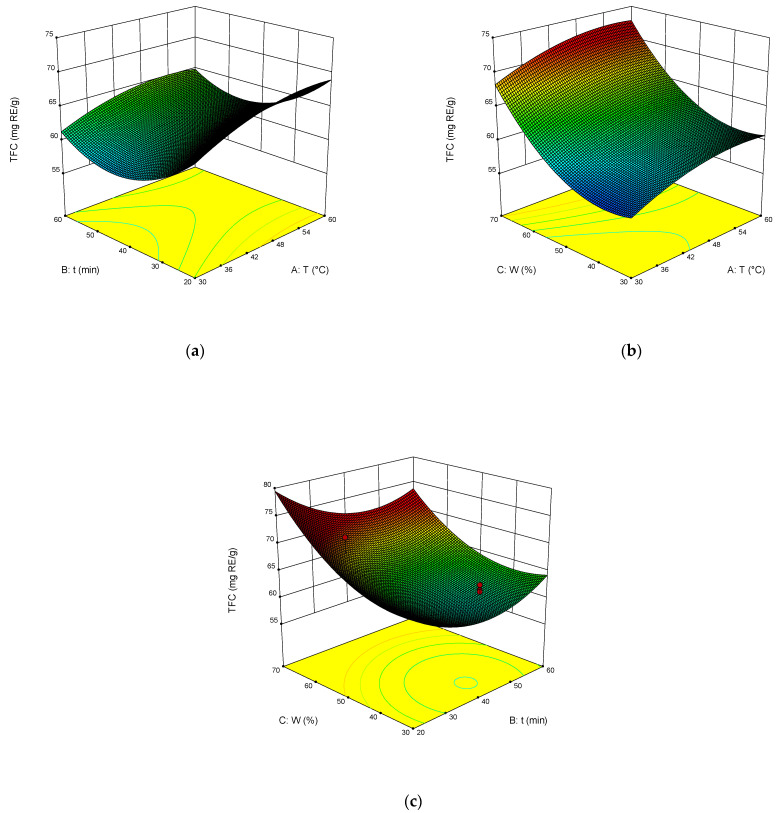
Response contour plots (**a**–**c**) showing the extraction temperature (A), extraction time (B), and water content (C) effect on the extraction yield of TFC.

**Figure 6:**
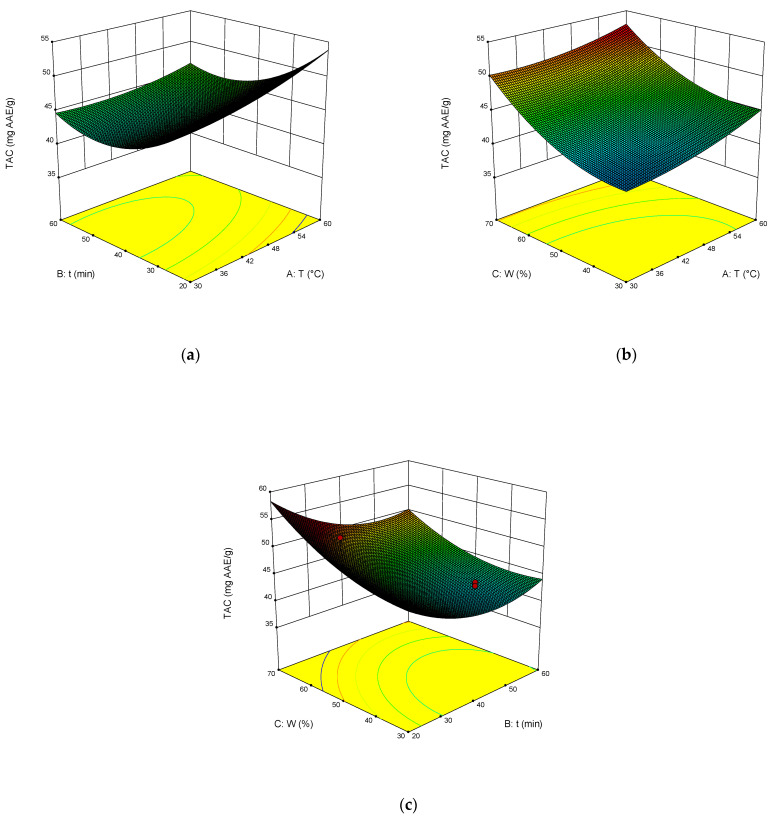
Response contour plots (**a**–**c**) showing the extraction temperature (A), extraction time (B), and water content (C) effect on the extraction yield of TAC.

**Figure 7:**
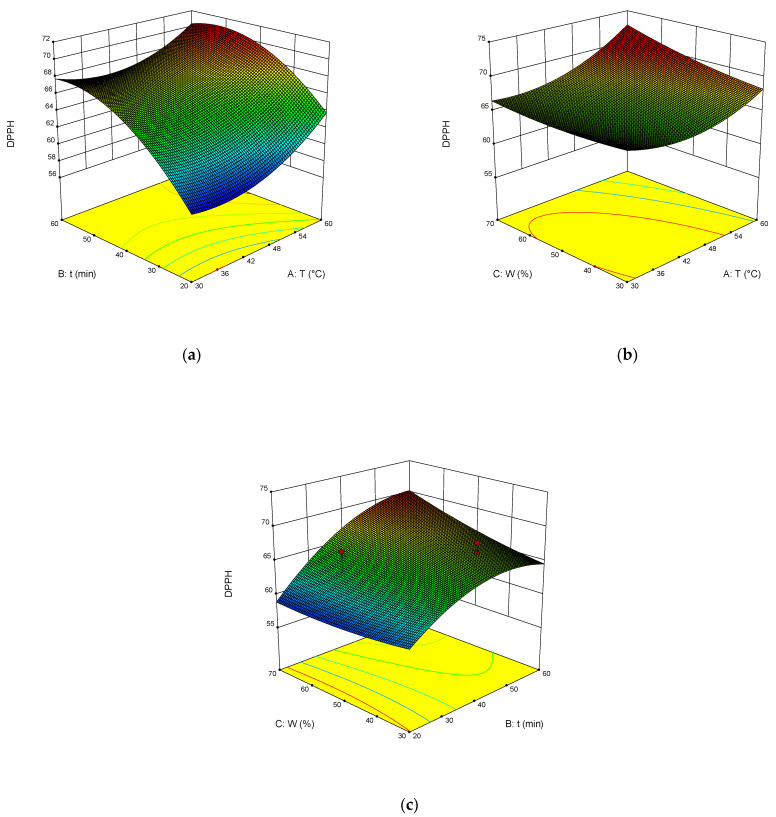
Response contour plots (**a**–**c**) showing the extraction temperature (A), extraction time (B), and water content (C) effect on the extraction yield of DPPH.

The dependence of TPC, TFC, TAC, and DPPH on the temperature, time of extraction, and water content in NADES was illustrated in the response surface contour plots generated by the model for the extraction. They are presented in Figure 4, Figure 5, Figure 6 and Figure 7. The response surfaces of TPC, TFC, and TAC displayed similar behaviors, while DPPH showed distinct response profiles.

Increases in the temperature and water content increased polyphenol yields. A similar trend was observed for flavonoid yields. Moreover, the higher the temperature, the greater the TAC and DPPH values, which may seem somewhat strange since some substances that exhibit antioxidant properties are thermolabile.

From Equations (3)–(6), the optimal conditions of extraction were found: temperature 58 °C, time of extraction 35 min, and 70 wt% water. At these conditions, TPC reached 301 mg GAE mL^−1^, TFC reached 74 mg RE mL^−1^, TAC reached 54 mg AAE mL^−1^, and DPPH reached 70%.

Table 5 shows that the composition of the ethanol extract was very different from other extracts, while the NADES extracts were similar in composition to the aqueous extracts. The GC-MS chromatograms and peaks identification for each solvent are also presented in the Supplementary Materials (S1–S6).

The main components of the ethanol extract were 5-(hydroxymethyl)-2-furancarboxaldehyde, 1,2,3-benzenetriol, octadecanal, and tricosanol. The main components of water- and NADES-based extracts were bis(2-ethylhexyl) ester of adipic acid, 3-(octadecyloxy)propyl ester of oleic acid, α-farnesene, and squalene. For the water extracts and NADES choline chloride + malic acid, the identical components were 2-hexanone, 3,3-dimethyl-2-hexanone, 4-butoxy-2-butanone, and 3-hexen-2-one. Moreover, these components were not extracted by the other studied solvents. Specific components of the extract based on NADES choline chloride + citric acid were aliphatic hydrocarbons such as heptadecane, hexadecane, and eicosane.

Some of the volatile substances found in this work were previously found in extracts and essential oils of *C. angustifolium*. Thus, in [1], the presence of myristic, lauric, palmitic, and oleic acids was mentioned. Palmitic, oleic, and stearic acids were found in *C. angustifolium* samples from the Ternopil region (Ukraine) [3]. Benzeneacetaldehyde was found in ethanolic extracts of *C. angustifolium* collected in Lithuania, but no other components common to this work have been described [4]. Aliphatic hydrocarbons (heptadecane, hexadecane, eicosane, nonacosane, and heneicosane), and aliphatic alcohols (phytol, tricosanol, and hexacosanol) were identified in [6] in *C. angustifolium* collected in the Central Siberian Botanical Garden, Siberian Branch, Russian Academy of Sciences, as in this work.

Thus, it should be noted that plant samples of *C. angustifolium* from different regions differ in composition, and a detailed phytochemical analysis of the volatile components as well as the influence of growing conditions on the chemical composition of this plant needs more detailed study.

## 4. Conclusions

Bioactive components were, for the first time, extracted with the NADES from *Chamaenerion angustifolium* (L.) Scop. (fireweed) growing in the Kola Peninsula. Solvents were formed with choline chloride and malonic, malic, tartaric, and citric acids, and with common solvents, such as water and 90% *v*/*v* ethanol.

The kinetics of the extraction processes were studied, and the parameters of the second-order reaction kinetics model for each solvent were obtained. It was shown that in 60 min the extraction of polyphenols and flavonoids almost reaches equilibrium. The values of the antioxidant and antiradical activity reach a plateau within a shorter time, which indicates a high rate of extraction of substances responsible for these parameters.

It was found that NADES choline chloride + citric acid is the most effective solvent for the extraction of biologically active compounds. The extraction conditions were optimized using BBD. The optimal conditions are: temperature 58 °C, extraction time 35 min, and 70 wt% water.

A GC-MS analysis of the extracts obtained using various solvents was performed. It was found that the compositions of the NADES-based extracts were close to the aqueous extracts. The main components of the NADES-based extracts were bis(2-ethylhexyl) ester of adipic acid, 3-(octadecyloxy)propyl ester of oleic acid, α -farnesene, and squalene.

The obtained results expand our knowledge about the use of NADES for the extraction of biologically active compounds from various types of plant raw materials and can potentially be useful for the development of environmentally friendly methods for the production of natural biologically active additives and pharmaceuticals.

## Figures and Tables

**Table 1:** Box–Behnken design of experiment parameters.

Parameter	Symbol	Levels with Code		
		−1	0	1
Temperature (°C)	A	30	45	60
Extraction time (min)	B	20	40	60
Water addition (wt%)	C	30	50	70

**Table 2:** Parameters of second-order kinetic model applied for TPC, TFC, TAC, and DPPH; “(Eq)” means “equilibrium values”; k: rate constant.

Solvent	TPC (Eq)	k_TPC_ × 10^3^ g·mg^−1^·min^−1^	TFC (Eq)	k_TFC_ × 10^3^ g·mg^−1^·min^−1^	TAC (Eq) g·mg^−1^·min^−1^	k_TAC_ × 10^3^ g·mg^−1^·min^−1^	DPPH (Eq)	k_DPPH_ × 10^3^ %^−1^ min^−1^
EtOH	169.1	2.5	92.0	2.7	41.5	10.1	31.4	55.8
Water	188.8	1.0	89.8	1.4	35.8	144.6	40.4	155.6
MA	212.5	1.2	74.2	1.9	34.7	13.2	59.0	14.8
Mal	246.8	0.5	78.6	1.1	29.1	9.0	56.0	2.3
Tar	309.0	0.3	67.2	1.9	44.7	1.8	65.6	186.4
CA	314.9	0.6	76.3	3.6	56.9	3.1	70.8	34.4

**Table 3:** Results of Tukey’s HSD test of estimation a statistically significant difference at *p* ≤ 0.05 between solvents for TPC, TFC, TAC, and DPPH.

Group 1	Group 2	TPC			TFC			TAC			DPPH		
		q-Stat	*p*-Value	Significance	q-Stat	*p*-Value	Significance	q-Stat	*p*-Value	Significance	q-Stat	*p*-Value	Significance
Tar	CA	9.29	<0.0001	Yes	6.65	0.005	Yes	7.92	0.001	Yes	1.70	0.827	No
Tar	MA	20.74	<0.0001	Yes	3.28	0.258	No	5.93	0.012	Yes	4.07	0.110	No
Tar	Mal	12.84	<0.0001	Yes	1.56	0.870	No	9.94	<0.0001	Yes	9.14	<0.0001	Yes
Tar	EtOH	33.45	<0.0001	Yes	4.29	0.086	No	3.47	0.213	No	15.92	<0.0001	Yes
Tar	Water	33.05	<0.0001	Yes	1.05	0.972	No	5.61	0.018	Yes	11.79	<0.0001	Yes
CA	MA	30.03	<0.0001	Yes	3.37	0.236	No	13.86	<0.0001	Yes	2.37	0.571	No
CA	Mal	22.13	<0.0001	Yes	5.09	0.033	Yes	17.87	<0.0001	Yes	7.43	0.002	Yes
CA	EtOH	42.74	<0.0001	Yes	2.37	0.571	No	11.39	<0.0001	Yes	14.22	<0.0001	Yes
CA	Water	42.34	<0.0001	Yes	7.70	0.002	Yes	13.54	<0.0001	Yes	10.09	<0.0001	Yes
MA	Mal	7.90	<0.0001	Yes	1.72	0.822	No	4.01	0.118	No	5.07	0.034	Yes
MA	EtOH	12.71	<0.0001	Yes	1.00	0.977	No	2.46	0.533	No	11.85	<0.0001	Yes
MA	Water	12.31	<0.0001	Yes	4.33	0.082	No	0.32	1.000	No	7.72	0.002	Yes
Mal	EtOH	20.62	<0.0001	Yes	2.72	0.433	No	6.47	0.006	Yes	6.78	0.004	Yes
Mal	Water	20.21	<0.0001	Yes	2.61	0.475	No	4.33	0.082	No	2.66	0.458	No
EtOH	Water	0.41	1.000	No	5.33	0.025	Yes	2.14	0.663	No	4.13	0.103	No

**Table 4:** Model summary and analysis of variance (ANOVA) of TPC, TFC, TAC, and DPPH of the *Chamaenerion angustifolium* (L.) Scop. leaf extracts.

Source	Sum of Squares				Mean Square				*F*-Value				*p*-Value			
	TPC	TFC	TAC	DPPH	TPC	TFC	TAC	DPPH	TPC	TFC	TAC	DPPH	TPC	TFC	TAC	DPPH
Model	1804.39	305.55	146.67	98.67	200.49	33.95	16.30	10.96	6.71	3.72	2.57	1.25	0.0101	0.0487	0.1131	0.3926
A-T	246.49	18.06	11.90	16.40	246.49	18.06	11.90	16.40	8.24	1.98	1.88	1.87	0.0240	0.2025	0.2127	0.2136
B-t	0.3309	6.88	18.88	37.98	0.3309	6.88	18.88	37.98	0.0111	0.7533	2.98	4.33	0.9192	0.4142	0.1279	0.0759
C-W	622.28	81.49	42.40	3.22	622.28	81.49	42.40	3.22	20.81	8.92	6.69	0.3675	0.0026	0.0203	0.0361	0.5635
AB	5.29	0.4225	2.56	2.25	5.29	0.4225	2.56	2.25	0.1769	0.0463	0.4042	0.2567	0.6866	0.8358	0.5451	0.6279
AC	0.0625	0.0625	0.7225	4.62	0.0625	0.0625	0.7225	4.62	0.0021	0.0068	0.1141	0.5274	0.9648	0.9364	0.7455	0.4913
BC	22.56	5.52	5.76	10.89	22.56	5.52	5.76	10.89	0.7546	0.6046	0.9094	1.24	0.4138	0.4623	0.3720	0.3018
A^2^	8.85	10.81	3.47	20.52	8.85	10.81	3.47	20.52	0.2961	1.18	0.5475	2.34	0.6032	0.3126	0.4834	0.1699
B^2^	156.67	83.29	55.56	34.62	156.67	83.29	55.56	34.62	5.24	9.12	8.77	3.95	0.0559	0.0194	0.0211	0.0872
C^2^	394.13	75.96	25.43	1.31	394.13	75.96	25.43	1.31	13.18	8.32	4.01	0.1493	0.0084	0.0235	0.0851	0.7107
Residual	209.30	63.94	44.33	61.35	29.90	9.13	6.33	8.76								
Lack of Fit	80.24	53.55	29.88	51.74	26.75	17.85	9.96	17.25	0.8289	6.87	2.76	7.18	0.5431	0.0467	0.1761	0.0435
Pure Error	129.06	10.39	14.45	9.61	32.27	2.60	3.61	2.40								
Cor Total	2013.69	369.48	191.00	160.02												

**Table 5:** Chemical components of volatile compounds from extracts of *Chamaenerion angustifolium* (L.) Scop. analyzed by GC-MS.

Peak RT	Compound Detected	Peak Area (%)					
		EtOH	Water	MA	Mal	Tar	CA
3.63	Methyl-cyclohexane		4.02		5.73		
3.77	2-methyl-1,3-pentanediol		2.7				
4.36	Tetrahydro-2-furanmethanol					1.18	
4.65	2-hexanone		2.65		3.84	1.17	
5.26	Furfural	1.45					
5.56	Maleic anhydride		3.93			1.01	
6.23	2,2-dimethyl-tetrahydrofuran					2.86	
6.23	3,3-dimethyl-2-hexanone		9.39		18.8		
6.43	3-Hydroxy-3-methylvaleric acid					1.71	
6.43	4-Butoxy-2-butanone		6.3		11.61		
6.91	1-acetyl-1,2-epoxy-cyclopentane					2.11	
6.92	3-Hexen-2-one		6.34		15.55		
7.22	Caproic acid, ethyl ester			0.77			0.25
7.35	Acetic acid, hexyl ester		3.04	1.94	2.65	1.28	0.59
7.46	Tetrahydro-6-methyl-2H-pyran-2-one						0.05
7.51	2-ethyl-1-hexanol			0.13			
7.56	D-Limonene						0.12
7.71	Benzeneacetaldehyde			0.52			
8.59	2,3-dihydro-3,5-dihydroxy-6-methyl-4H-Pyran-4-one	1.41					
8.96	Caprylic acid, ethyl ester		1.59	1.24		1.52	0.6
8.96	5-ethyl-2,2,3-trimethyl-heptane				1.37		
9.24	5-(hydroxymethyl)-2-furancarboxaldehyde	10.43		3.9			
9.54	1-Decanol		1.54				
10.32	1,2,3-Benzenetriol	19.81		0.79			
10.35	Caproic acid, hexyl ester			0.34		0.69	
11.02	2,6-bis(1,1-dimethylethyl)-2,5-cyclohexadiene-1,4-dione				2.25		
11.10	Heptadecane						2.96
11.16	5-(1,5-dimethyl-4-hexenyl)-2-methyl-1,3-cyclohexadiene			2.31	3.01	7.31	2.16
11.19	α-Farnesene		11.69	6.29	7.95	13.42	4.46
11.38	Hexadecane						1.99
11.45	Lauric acid	0.34					
12.40	8-methyl-heptadecane					6.12	3.78
12.40	Heneicosane					2.82	
12.62	Myristic acid	1.39					
12.63	Eicosane						7.45
13.69	Palmitic acid	4.04	6.41	12.6			2.51
13.77	Nonacosane					4.5	8.62
13.79	Phthalic acid, bis(2-methylpropyl) ester					4.32	1.45
13.87	Palmitic acid, ethyl ester			0.79			
14.02	Isopropyl Palmitate			0.84		2.75	0.58
14.47	Phytol	1.02					
14.58	Oleic Acid			4.39			
14.60	Tetratetracontane					24.98	3.42
14.66	Stearic acid	0.45		8.03			
14.67	Adipic acid, mono(2-ethylhexyl)ester						1.16
15.74	Adipic acid, bis(2-ethylhexyl) ester		25.2	10.33	18.03	18.45	10.36
16.88	Octadecanal	17.64					
17.23	Oleic acid, 3-(octadecyloxy)propyl ester		12.34	4.52	8.52		2.72
17.25	Tricosanol	38.37					
18.23	Squalene		8.17	5.59	8.91		3.5
18.70	Hexacosanol	3.65					

## Data Availability

Not applicable.

## Supplementary Materials

The following supporting information can be downloaded at: https://www.mdpi.com/article/10.3390/molecules27134216/s1, S1. GC-MS chromatogram of ethanolic extract of *Chamaenerion angustifolium* (L.) Scop. and the table of peak identification; S2. GC-MS chromatogram of water extract of *Chamaenerion angustifolium* (L.) Scop. and the table of peak identification; S3. GC-MS chromatogram of extract of *Chamaenerion angustifolium* (L.) Scop. based on NADES chloride choline + malonic acid, and the table of peak identification; S4. GC-MS chromatogram of extract of *Chamaenerion angustifolium* (L.) Scop. based on NADES chloride choline + malic acid, and the table of peak identification; S5. GC-MS chromatogram of extract of *Chamaenerion angustifolium* (L.) Scop. based on NADES chloride choline + tartaric acid, and the table of peak identification; S6. GC-MS chromatogram of extract of *Chamaenerion angustifolium* (L.) Scop. based on NADES chloride choline + citric acid, and the table of peak identification.
