# Supplementary material for: Extraction of Bioactive Components from Chamaenerion angustifolium (L.) Scop. with Choline Chloride and Organic Acids Natural Deep Eutectic Solvents
Source: Molecules. 2022 Jun 30;27(13):4216. doi: 10.3390/molecules27134216 (PMC9268342; doi:10.3390/molecules27134216)
Supplement: Supplementary file 1 [file molecules-27-04216-s001.zip › molecules-1776264-supplementary.pdf]

Extraction of Bioactive Components from Chamaenerion angustifolium (L.) Scop. with Choline Chloride and Organic Acids Natural Deep Eutectic Solvents

Nikita Tsvetov [1,*], Elena Pasichnik[2], Anna Korovkina[3], and Alevtina Gosteva [1]

[1] I.V. Tananaev Institute of Chemistry and Technology of Rare Elements and Mineral Raw Materials - Subdivision of the Federal Research Centre «Kola Science Centre of the Russian Academy of Sciences», Akademgorodok 26a, Apatity, Russia; tsvet.nik@mail.ru; angosteva@list.ru
[2] Murmansk State Technical University, Akademgorodok 50a, Apatity, Russia; wlondr@yandex.ru
[3] Federal Research Centre "Kola Science Centre of the Russian Academy of Sciences", Laboratory of medical and biological technologies, Fersmana str. 14, Apatity, Russia; dokktorr@list.ru
* Correspondence: tsvet.nik@mail.ru

Supplementary materials

S1. GC-MS chromatogram of ethanolic extract of *Chamaenerion angustifolium* (L.) Scop. and the table of peak identification.

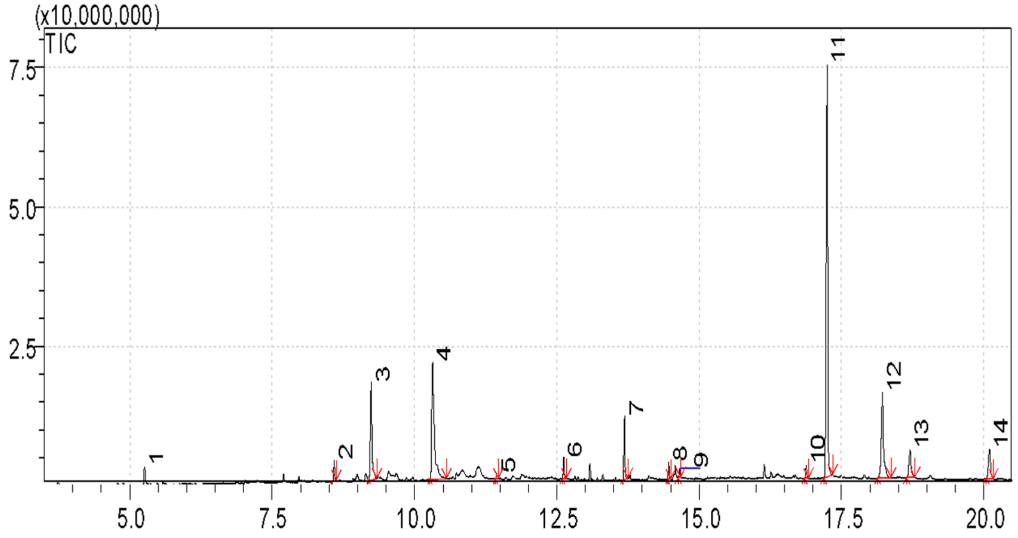

№	Peak area (%)	Component
1	1.45	Furfural
2	1.41	4H-Pyran-4-one, 2,3-dihydro-3,5-dihydroxy-6-methyl-
3	10.43	2-Furancarboxaldehyde, 5-(hydroxymethyl)-
4	19.81	1,2,3-Benzenetriol
5	0.34	Dodecanoic acid
6	1.39	Tetradecanoic acid
7	4.04	n-Hexadecanoic acid
8	1.02	Phytol
9	0.45	Octadecanoic acid
10	0.99	Octadecanal
11	38.37	1-Tricosanol
12	12.29	Not identified
13	3.65	1-Hexacosanol
14	4.36	Not identified

S2. GC-MS chromatogram of water extract of *Chamaenerion angustifolium* (L.) Scop. and the table of peak identification.

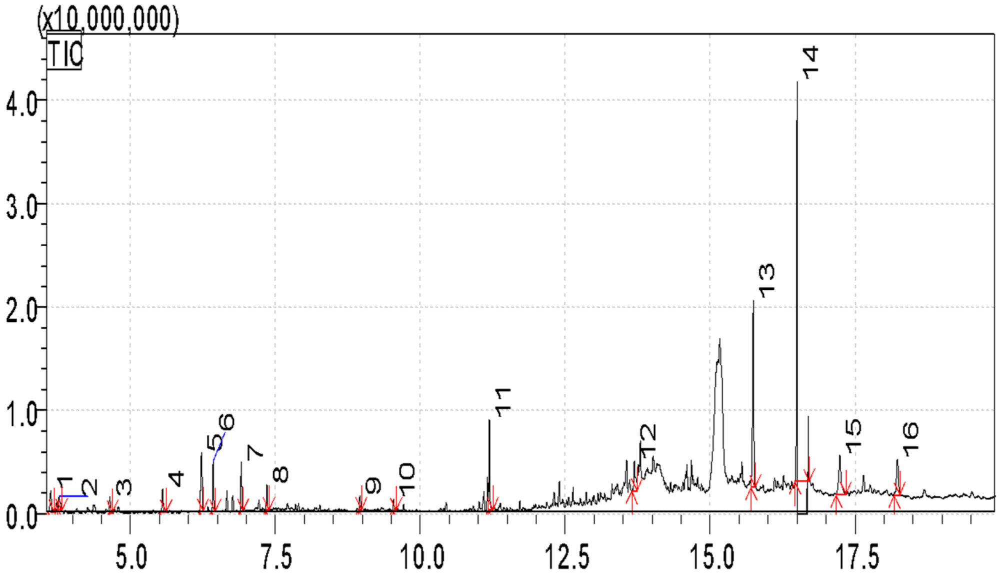

№	Peak area (%)	Component
1	4.02	Cyclohexane, methyl-
2	2.70	1,3-Pentanediol, 2-methyl-
3	2.65	2-Hexanone
4	3.93	Maleic anhydride
5	9.39	2-Hexanone, 3,3-dimethyl-
6	6.30	4-Butoxy-2-butanone
7	6.34	3-Hexen-2-one
8	3.04	Acetic acid, hexyl ester
9	1.59	Octanoic acid, ethyl ester
10	1.54	1-Decanol
11	11.69	.alpha.-Farnesene
12	6.41	n-Hexadecanoic acid
13	25.20	Hexanedioic acid, bis(2-ethylhexyl) ester
14	5.31	Not identified
15	12.34	9-Octadecenoic acid, 1,2,3-propanetriyl ester, (E,E,E)-
16	8.17	Squalene

S3. GC-MS chromatogram of extract of *Chamaenerion angustifolium* (L.) Scop. based on NADES chloride choline + malonic acid, and the table of peak identification.

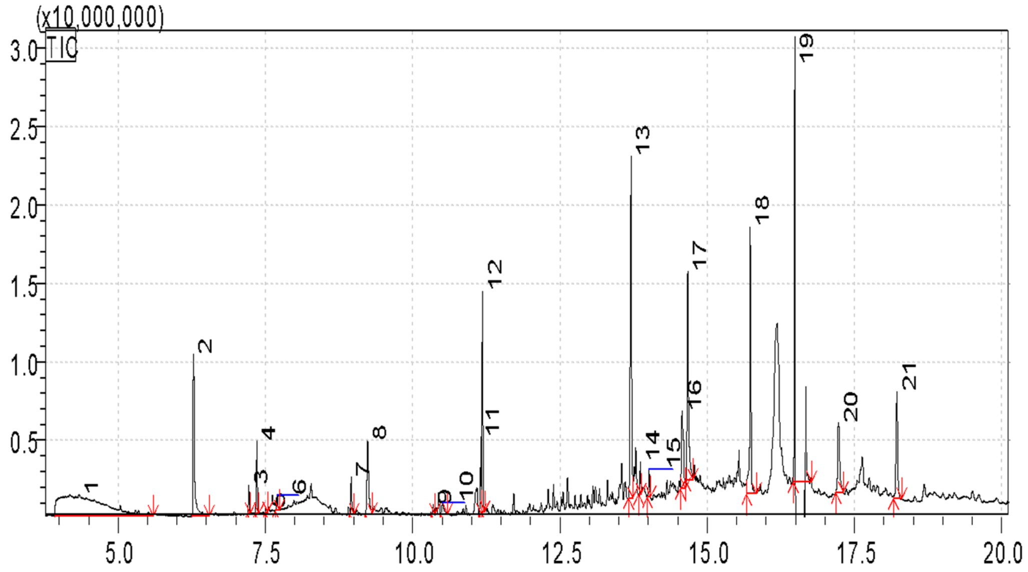

№	Peak area (%)	Component
1	28.26	N,N-Dimethyl-2-aminoethanol
2	7.99	Acetic acid, 2-(dimethylamino)ethyl ester
3	0.77	Hexanoic acid, ethyl ester
4	1.94	Acetic acid, hexyl ester
5	0.13	1-Hexanol, 2-ethyl-
6	0.52	Benzeneacetaldehyde
7	1.24	Octanoic acid, ethyl ester
8	3.90	2-Furancarboxaldehyde, 5-(hydroxymethyl)-
9	0.34	Hexanoic acid, hexyl ester
10	0.79	1,2,3-Benzenetriol
11	2.31	1,3-Cyclohexadiene, 5-(1,5-dimethyl-4-hexenyl)-2-methyl-,
12	6.29	.alpha.-Farnesene
13	12.60	n-Hexadecanoic acid
14	0.79	Hexadecanoic acid, ethyl ester
15	0.84	Isopropyl Palmitate
16	4.39	Oleic Acid
17	8.03	Not identified
18	10.33	Hexanedioic acid, bis(2-ethylhexyl) ester
19	1.57	Not identified
20	4.52	Oleic acid, 3-(octadecyloxy)propyl ester
21	5.59	Squalene

S4. GC-MS chromatogram of extract of *Chamaenerion angustifolium* (L.) Scop. based on NADES chloride choline + malic acid, and the table of peak identification.

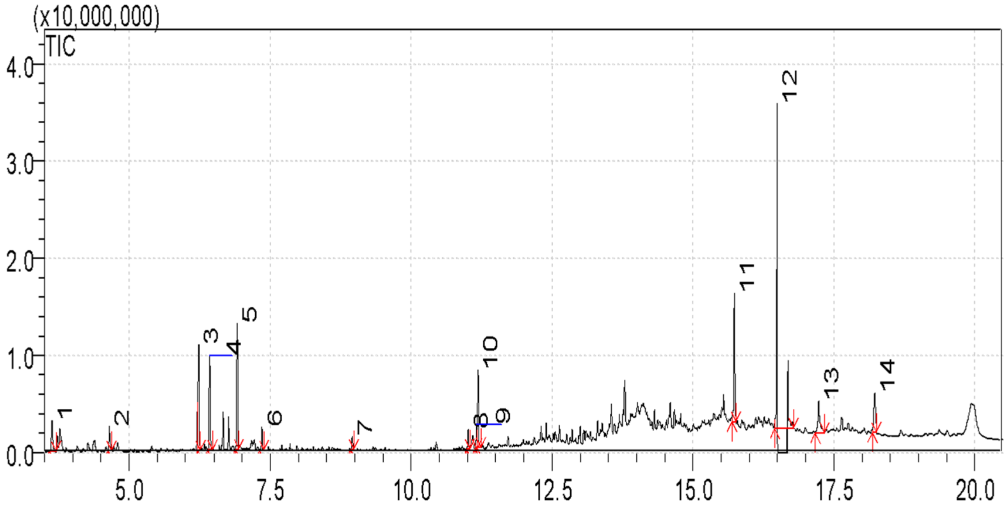

№	Peak area (%)	Component
1	5.73	Cyclohexane, methyl-
2	3.84	2-Hexanone, 4-hydroxy-3-propyl-
3	18.8	2-Hexanone, 3,3-dimethyl-
4	11.61	4-Butoxy-2-butanone
5	15.55	3-Hexen-2-one
6	2.65	Acetic acid, hexyl ester
7	1.37	Heptane, 5-ethyl-2,2,3-trimethyl-
8	2.25	2,5-Cyclohexadiene-1,4-dione, 2,6-bis(1,1-dimethylethyl)-
9	3.01	1,3-Cyclohexadiene, 5-(1,5-dimethyl-4-hexenyl)-2-methyl
10	7.95	.alpha.-Farnesene
11	18.03	Hexanedioic acid, bis(2-ethylhexyl) ester
12	8.22	Not identified
13	8.52	Oleic acid, 3-(octadecyloxy)propyl ester
14	8.91	Squalene

S5. GC-MS chromatogram of extract of *Chamaenerion angustifolium* (L.) Scop. based on NADES chloride choline + tartaric acid, and the table of peak identification.

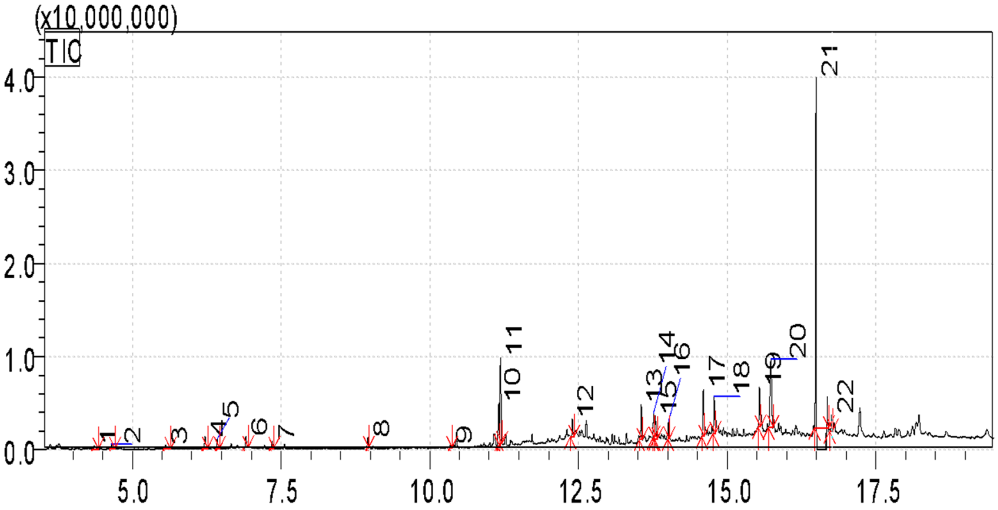

№	Peak area (%)	Component
1	1.18	2-Furanmethanol, tetrahydro-
2	1.17	2-Hexanone, 4-hydroxy-3-propyl-
3	1.01	Maleic anhydride
4	2.86	Tetrahydrofuran, 2,2-dimethyl-
5	1.71	3-Hydroxy-3-methylvaleric acid
6	2.11	Cyclopentane, 1-acetyl-1,2-epoxy-
7	1.28	Acetic acid, hexyl ester
8	1.52	Octanoic acid, ethyl ester
9	0.69	Hexanoic acid, hexyl ester
10	7.31	1,3-Cyclohexadiene, 5-(1,5-dimethyl-4-hexenyl)-2-methyl-,
11	13.42	.alpha.-Farnesene
12	2.82	Heneicosane
13	6.12	Heptadecane, 8-methyl-
14	4.50	Nonacosane
15	4.32	1,2-Benzenedicarboxylic acid, bis(2-methylpropyl) ester
16	2.75	Isopropyl Palmitate
17	8.05	Not identified
18	6.28	Not identified
19	6.90	Not identified
20	18.45	Hexanedioic acid, bis(2-ethylhexyl) ester
21	1.80	Not identified
22	3.75	Tetracosane

S6. GC-MS chromatogram of extract of *Chamaenerion angustifolium* (L.) Scop. based on NADES chloride choline + citric acid, and the table of peak identification.

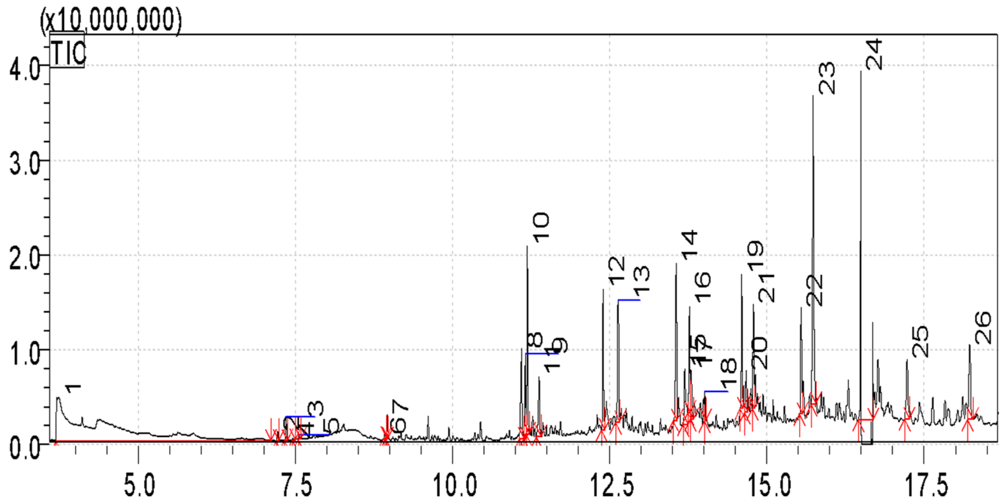

№	Peak area (%)	Component
1	40.98	N,N-Dimethyl-2-aminoethanol
2	0.25	Hexanoic acid, ethyl ester
3	0.59	Acetic acid, hexyl ester
4	0.05	2H-Pyran-2-one, tetrahydro-6-methyl-
5	0.12	D-Limonene
6	0.07	Hexanoic acid, butyl ester
7	0.53	Octanoic acid, ethyl ester
8	2.96	Heptadecane
9	2.16	1,3-Cyclohexadiene, 5-(1,5-dimethyl-4-hexenyl)-2-methyl-,
10	4.46	.alpha.-Farnesene
11	1.99	Hexadecane
12	3.78	Heptadecane, 8-methyl-
13	3.34	Eicosane
14	4.11	Eicosane
15	2.51	n-Hexadecanoic acid
16	2.99	Tetracosane
17	1.45	1,2-Benzenedicarboxylic acid, butyl 8-methylnonyl ester
18	0.58	Isopropyl Palmitate
19	3.42	Tetratetracontane
20	1.16	Hexanedioic acid, mono(2-ethylhexyl)ester
21	2.89	Tetracosane
22	2.74	Tetracosane
23	10.36	Hexanedioic acid, bis(2-ethylhexyl) ester
24	0.29	Not identified
25	2.72	9-Octadecenoic acid, 1,2,3-propanetriyl ester, (E,E,E)-
26	3.50	Squalene
